# Solar light driven atomic and electronic transformations in a plasmonic Ni@NiO/NiCO_3_ photocatalyst revealed by ambient pressure X-ray photoelectron spectroscopy†

**DOI:** 10.1039/d4cy00204k

**Published:** 2024-04-22

**Authors:** Manoj Kumar Ghosalya, Parisa Talebi, Harishchandra Singh, Alexander Klyushin, Esko Kokkonen, Mohammed Alaoui Mansouri, Marko Huttula, Wei Cao, Samuli Urpelainen

**Affiliations:** a Nano and Molecular Systems Research Unit, University of Oulu FIN-90014 Finland manoj.ghosalya@oulu.fi; b MAX IV Laboratory, Lund University Box 118 Lund 22100 Sweden

## Abstract

This work employs ambient pressure X-ray photoelectron spectroscopy (APXPS) to delve into the atomic and electronic transformations of a core–shell Ni@NiO/NiCO_3_ photocatalyst – a model system for visible light active plasmonic photocatalysts used in water splitting for hydrogen production. This catalyst exhibits reversible structural and electronic changes in response to water vapor and solar simulator light. In this study, APXPS spectra were obtained under a 1 millibar water vapor pressure, employing a solar simulator with an AM 1.5 filter to measure spectral data under visible light illumination. The *in situ* APXPS spectra indicate that the metallic Ni core absorbs the light, exciting plasmons, and creates hot electrons that are subsequently utilized through hot electron injection in the hydrogen evolution reaction (HER) by NiCO_3_. Additionally, the data show that NiO undergoes reversible oxidation to NiOOH in the presence of water vapor and light. The present work also investigates the contribution of carbonate and its involvement in the photocatalytic reaction mechanism, shedding light on this seldom-explored aspect of photocatalysis. The APXPS results highlight the photochemical reduction of carbonates into –COOH, contributing to the deactivation of the photocatalyst. This work demonstrates the APXPS efficacy in examining photochemical reactions, charge transfer dynamics and intermediates in potential photocatalysts under near realistic conditions.

## Abstract 2

This work employs ambient pressure X-ray photoelectron spectroscopy (APXPS) to delve into the atomic and electronic transformations of a core–shell Ni@NiO/NiCO_3_ photocatalyst – a model system for visible light active plasmonic photocatalysts used in water splitting for hydrogen production.

## Graphical abstract

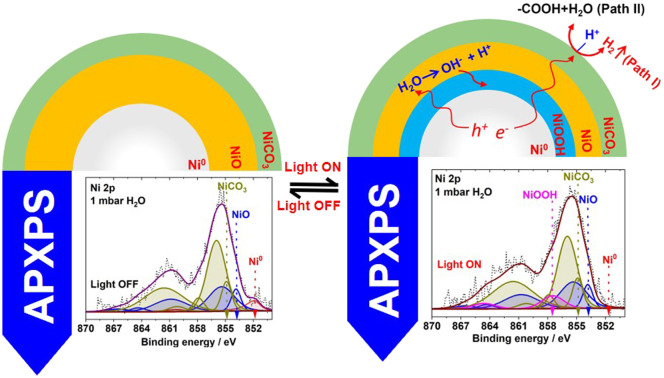

## Introduction

Poor photocatalytic efficiency and stability of photocatalysts have so far impeded their feasibility for industrial-level hydrogen production.^1–3^ The imperative for progress persists, primarily due to the intricate nature of the catalysis process during operation. Photocatalysis is a multistep process involving photon absorption, charge excitation and separation, reactant adsorption, product desorption, and redox reactions on photocatalyst surfaces.^4^ To accelerate the progress in the HER, it is crucial to gain a comprehensive insight into the atomistic-level intricacies inherent to the photocatalytic process, particularly under *in situ* conditions. The conventional *ex situ* characterization techniques such as TEM, UHV-XPS, XRD and UV-visible spectroscopy can offer insights into the morphology, crystal structure, oxidation state, and optical and chemical properties of a photocatalyst. However, these *ex situ* measurements, in most cases, can only distinguish the pre- and post-reaction electronic and chemical characteristics of a photocatalyst. They lack the ability to analyze short-lived reaction intermediates, active catalyst sites, functional groups, and charge transfer dynamics, which can be observed only under *operando* photocatalytic conditions.^5,6^ Another complication with *ex situ* measurements is the contamination of the photocatalyst surface during spectroscopic measurements, storage, or transportation of catalysts. This contamination could significantly alter the spectroscopic fingerprints of surface groups and the chemical and electronic states of active sites potentially leading to false interpretations and an incomplete picture of the state of the catalyst. Hence, combining the photocatalyst's performance with a mechanistic understanding under *operando* reaction conditions is crucial for the rational and systematic design of a more advanced photocatalyst.^7–9^ Ambient pressure X-ray photoelectron spectroscopy (APXPS) is among other chemical analysis techniques used to study surfaces and interfaces under *in situ* conditions.^10–12^ The unique advantage of APXPS over other spectroscopic techniques is its surface sensitivity. Photochemical processes happen at the interface of water and the photocatalyst. Due to its high surface sensitivity, APXPS is a suitable technique to study the chemical and electronic changes including reaction intermediates, active catalyst sites, and functional groups at the photocatalyst–water interface in real time.^13,14^ Furthermore, APXPS can be used to elucidate the charge transfer dynamics^15^ and specific chemical species or elements acting as charge donors or acceptors during photocatalytic reactions.^16^

This work is dedicated to exploring the photocatalytic mechanism of a recently discovered Ni-based plasmonic photocatalyst *i.e.*, Ni@NiO/NiCO_3_.^17,18^ As noted in previous work, this model system can have advantages over semiconductor-based systems and conventional plasmonic photocatalysts which are affected by high reagent costs, complex material synthesis, and limited efficiency. Briefly, these reports (ref. 17 and 18) reveal that the Ni^0^ core of the catalyst can absorb light through a phenomenon known as surface plasmon resonance (SPR), which leads to the generation of so-called hot electrons.^19,20^ These hot electrons can be injected into the nanoparticle shell and further accepted by the O atoms in NiCO_3_. Additionally, earlier studies^18,21^ highlighted NiCO_3_ as a pivotal surface for hydrogen ion adsorption, facilitating hydrogen ion reduction and ultimately generating molecular hydrogen. However, the previous investigations do not provide comprehensive experimental insights into the intermediate species of photochemical transformations related to water oxidation and reduction in the photocatalyst, due to the light absorption. Furthermore, the long-term stability and the chemical degradation mechanism of the photocatalyst, especially the carbonates under operational conditions, stemming from potential side photochemical reactions, have not been considered previously. Moreover, though structurally and chemically different, there are also a few known plasmonic Ni-based photocatalysts such as Ni@C@TiO_2_, Ni@C-CdS, and Ni@Ni_2_P-g-C_3_N_4_ which have been reported so far.^22,23^ However, the understanding of the photocatalytic mechanism is still a major hurdle, and therefore a thorough understanding of such plasmonic photocatalysts is required towards their rational design.

By utilizing the aforementioned advantages of APXPS, the present study elucidates the mechanisms of photocatalytic water-splitting in a novel potential model system, *i.e.*, the Ni@NiO/NiCO_3_ photocatalyst,^17,18^ under solar simulator light illumination and dark conditions at 1 mbar water vapor pressure. The Ni 2p and O 1s spectra revealed that NiO underwent reversible oxidation to NiOOH, confirming that holes were utilized in water oxidation on NiO under solar light illumination. The C 1s spectra confirmed that H_2_ evolution occurred at the NiCO_3_ surface, along with the potential photochemical pathways responsible for the reduction of carbonates to –COOH. The photocatalytic reduction of carbonates to –COOH contributed to the gradual decrease in HER efficiency of Ni@NiO/NiCO_3_ over time. To identify patterns and subtle variations within the spectroscopic data, principal component analysis (PCA) was employed, providing a useful tool for understanding the behavior of Ni 2p, C 1s, and O 1s spectra under different reaction conditions.

## Experimental section

APXPS measurements were conducted at the APXPS branch of the SPECIES beamline at MAX IV Laboratory, Sweden.^24,25^ The SPECIES beamline is a soft X-ray beamline that provides X-ray photon energies ranging from 30 eV to 1500 eV. All XPS measurements presented in this work were performed with an ambient-pressure gas cell and a differentially pumped electron analyzer (SPECS, PHOIBOS 150 NAP) at the APXPS end station. This ambient pressure gas cell is built with the cell-in-cell concept where the reaction cell is incorporated into the UHV chamber. The ambient pressure cell is designed to perform chemical reactions at pressures up to 20 mbar and temperatures up to 600 °C. More details of the experimental setup are given elsewhere.^24,25^ As a part of *in situ* photocatalysis experiments, a solar simulator (Asahi HAL-320) was installed at the APXPS endstation.^26^ The solar simulator was equipped with an AM 1.5 filter, which allows light to pass through in spectral ranges between 350 and 1100 nanometers. The light from the solar simulator is directed at the sample through a fused silica viewport on the UHV chamber and passes through a sapphire window on the ambient pressure cell. The schematic layout of this setup and the solar simulator spectra recorded with both windows are shown elsewhere.^26^

The photocatalyst used in this work is commercially available Ni nanoparticles from Hongwu International. Due to possible oxidation and carbonation while the metal nanoparticles are mass produced, these Ni nanoparticles are transformed into a complex Ni@NiO/NiCO_3_ core@shell morphology.^27^ Detailed XPS data analysis suggests that the catalyst core consists of metallic Ni (Ni^0^) and the shell is made of NiO and NiCO_3_.^17^ Previous experiments using XRD show that Ni^0^ and NiO have a crystalline morphology, whereas NiCO_3_ is amorphous.^17^ The Ni@NiO/NiCO_3_ photocatalyst has been shown to be active toward water splitting and to produce hydrogen without any sacrificial agent under visible light illumination. However, the overall water splitting efficiency of the nanoparticles could be improved and optimized by further annealing them at different temperatures. Indeed, the Ni@NiO/NiCO_3_ photocatalyst morphology depends on the annealing time and temperature. In a previous study, a series of catalysts were prepared and tested for the HER. The amount of carbonate in the catalyst shell was shown to be positively correlated with the HER and could be tuned by vacuum annealing at different temperatures and for different durations. Catalysts with the highest carbonate content (44%) demonstrated the highest HER activity. Therefore, the most active Ni@NiO/NiCO_3_ annealed in a vacuum for two hours at 100 °C (N70–100/2 h) was chosen for the present work.^17^

Ni@NiO/NiCO_3_ photocatalyst samples for APXPS measurements were prepared by dispersing catalyst powder in water and drop-casting it onto gold foil. A thin catalyst layer was formed on the gold foil after the water had evaporated. After the preparation, the sample was mounted on a stainless-steel sample plate and introduced into the experimental chamber and the ambient pressure cell. All the APXPS system's viewports were covered with aluminum foil to protect the photocatalyst from external light during the measurements. First, the XPS spectra were recorded under UHV conditions (base pressure 1 × 10^−8^ mbar) with light illumination and in the dark. After the UHV characterization, the catalyst was exposed to 1 mbar water vapor through a precision leak valve. APXPS spectra were then measured with and without solar simulator illumination and the evolution of the Ni 2p, C 1s, and O 1s spectra was monitored. All core levels were measured with a step size of 0.05 eV using a 1 mm analyzer slit at a constant pass energy of 50 eV. By adjusting the X-ray photon energy, XPS spectra were acquired close to the interface of Ni and NiO/NiCO_3_ with the same kinetic energy (∼150 eV). The photon energies used to measure Ni 2p, C 1s and O 1s were 1000, 680, and 430 eV, respectively. The adventitious carbon peak (C-sp^3^) at 285 eV was used for the binding energy (BE) calibration for the O 1s and Ni 2p peaks.^28^ The fitting parameters are shown in Tables S1–S3 in the ESI.† The APXPS measurements were repeated several times to ensure the reproducibility of the data under *in situ* conditions. Spectra recorded under both dark and light illumination conditions were considered as one cycle. Each experimental cycle under UHV and *in situ* conditions entailed a duration of 114 minutes, with 56 minutes dedicated to dark conditions and an equal duration under light exposure, totaling 228 minutes for two complete cycles of *in situ* measurements and 114 minutes under UHV with light illumination and in the dark.

A principal component analysis (PCA) was conducted by using MATLAB on the APXPS spectra. PCA was used to examine statistical differences of the small variations between the measurements under different conditions in order to identify the specific variables that might contribute to these differences. It is possible to use this information to gain a better understanding of the physical processes underlying the behavior of the samples under different conditions.^29–31^ Furthermore, PCA can help to simplify the XPS data analysis process by reducing the dimensionality of the data and identifying the underlying chemical components that contribute to the complex spectrum. The data matrices for PCA included the Ni 2p XPS spectra recorded in the BE range of 850–895 eV into 451 points. In addition to the Ni data, PCA was also applied on the O 1s and C 1s data from 522 to 538.58 eV for 301 points and from 278.89 to 298.89 eV for 401 points, respectively.

In a separate experiment, the photocatalytic HER was evaluated on the given sample using a quartz container with dimensions of 90 mm in height and 35 mm in diameter, having a total volume of approximately 68 mL. 5 mg of catalyst was mixed in 25 mL of deionized water and sonicated for one minute. LED light sources integrated with a magnetic stirrer within the Perfect Light PCX50B photoreactor were employed for excitation. The produced H_2_ was quantified using an Agilent Micro 490 gas chromatograph (GC) that featured a column sensitive to hydrogen. To assess stability, the catalyst was subjected to a 10 hour test under continuous light exposure, with hydrogen production measured every 2 hours. Importantly, these measurements were conducted without the use of any cocatalysts or agents for electron/hole preservation.

## Results

### (a) Catalyst under light illumination and dark conditions under UHV

**Fig. 1:**
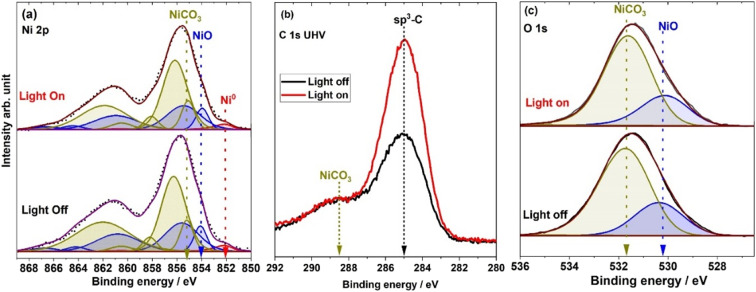
Core level spectra with light illumination ON (red) and OFF (black) under UHV conditions for (a) Ni 2p, (b) C 1s and (c) O 1s.

Fig. 1(a)–(c) depict the Ni 2p (a), C 1s (b) and O 1s (c) spectra acquired under UHV conditions under light illumination and in the dark. The Ni 2p spectrum contains several photoemission peaks corresponding to the different oxidation states of the different Ni species.^32^ The spectra were fitted using an asymmetric Lorentzian line shape (LA) after removing the Shirley background using casaXPS software. The BEs of the Ni species are taken from the work of Grosvenor *et al.*^33^ and Biesinger *et al.*^34,35^ However, in the present case, the BEs vary slightly from these published studies due to the different catalyst compositions.^36^ The Ni^0^ 2p main peak with two satellite peaks was observed at 852.1 eV whereas the NiO and NiCO_3_ 2p peaks are fitted at 854.1 and 855.1 eV, respectively, with four satellites each.^34,35^ The accuracy of peak BE calibration is ±0.2 eV. The BEs of the main peaks associated with Ni^0^, NiO, and NiCO_3_ are marked using dashed vertical arrows in the figures. For the best peak fit, the satellite peak energies and intensity ratios were kept constant with respect to the main peaks. The fitting parameters are shown in Table S1 in the ESI.† The satellite peaks at 855.6 and 856.2 eV, associated respectively with NiO and NiCO_3_, exhibit higher intensity compared to their respective main peak counterparts. This observation aligns with the findings by Soriano *et al.*,^37^ who elucidated that the BE of the main peak for surface Ni^+2^ ions in NiO shifts towards higher energy sites, contributing to the increased intensity of the satellite peak at 855.62 eV. As previously mentioned, Ni 2p spectra were recorded using a 1000 eV excitation energy and given that the thickness of NiO is only a few nanometers, the surface Ni^+2^ ions significantly influence the Ni 2p peaks associated with NiO. This contribution, combined with the effects of nonlocal screening,^37,38^ results in a significant increase in the intensity of the satellite peak associated with NiO compared to the main peak. Given that the thickness of NiCO_3_ is similar to the NiO thickness (Fig. 1a) and the used X-ray excitation energy is also comparable, the same concept can be applied to explain the higher intensity of the satellite peak associated with NiCO_3_.

The Ni 2p spectra recorded under UHV (Fig. 1b, ESI† Fig. S1) remain virtually unchanged, regardless of whether the spectra were recorded with light illumination or without. Fig. 1b shows the C 1s spectra under UHV conditions. Two clear peaks are observed centered at 285 eV corresponding to adventitious carbon (sp^3^-carbon) and 288.4 eV corresponding to NiCO_3_ carbon.^32^ When the solar simulator was turned ON, an increase in C 1s intensity at 285 eV was observed, corresponding to adventitious carbon. Since the solar simulator emits also IR radiation,^26^ the catalyst surface becomes heated and the sample temperature can reach up to a maximum of 90 °C according to the thermocouple reading on the sample plate. Under UHV, with light illumination, adventitious carbon accumulated on the catalyst surface most probably due to the high catalyst surface temperatures. In contrast, the carbonate peak does not change under light illumination. Similarly, no change is observed in the O 1s spectra with light illumination and without (Fig. 1c). The peaks at 530.2 and 531.5 eV correspond to NiO and NiCO_3_, respectively.^32^

### (b) Catalyst light illumination ON/OFF under 1 mbar water vapor

**Fig. 2:**
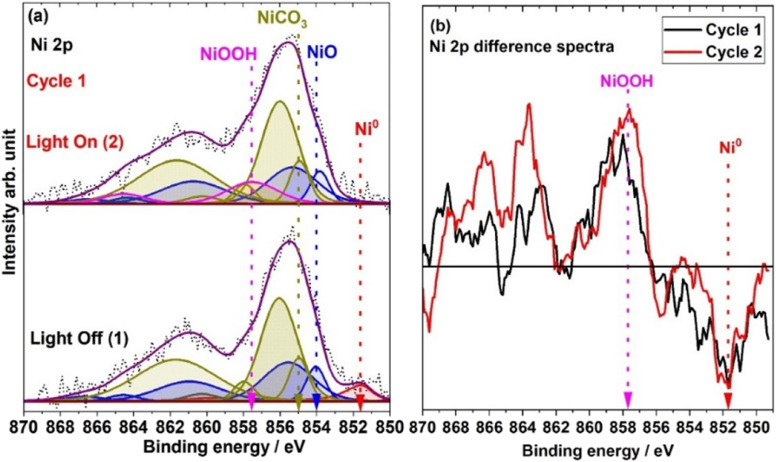
*In situ* XPS measurement of chemical state evolution of Ni during the photocatalytic reaction. (a) The spectra were recorded at 1 mbar H_2_O pressure under dark (bottom spectra) and light illumination (top spectra) conditions. (b) Ni 2p difference spectra for the two cycles (black and red color spectra represent cycles 1 and 2, respectively); the difference spectra are generated by subtracting the spectrum recorded under dark conditions from the spectrum recorded under solar simulator light illumination. The Savitzky–Golay smoothing process was applied on the Ni 2p spectra before calculating the difference spectra.^39^

Fig. 2 shows the Ni 2p spectra recorded under 1 mbar H_2_O under dark conditions and light illumination. Also, in this case an increase in the temperature of the catalyst surface was observed, reaching at most 45 °C under light illumination. The difference in temperature with respect to UHV experiments can be explained by the heat dissipation caused by the water vapor at 1 mbar pressure. The XPS measurements were repeated twice to ensure the reproducibility of the data. The presented data include only the first cycle of spectra recorded under light illumination and dark conditions, while the results from the second cycle are provided in ESI† Fig. S2. In both cycles, the XPS data are similar indicating that the results are reproducible. The bottom and top spectra in Fig. 2a were acquired without and with light illumination, respectively. The Ni 2p spectra recorded under dark conditions are very similar to the UHV spectra.

**Fig. 3:**
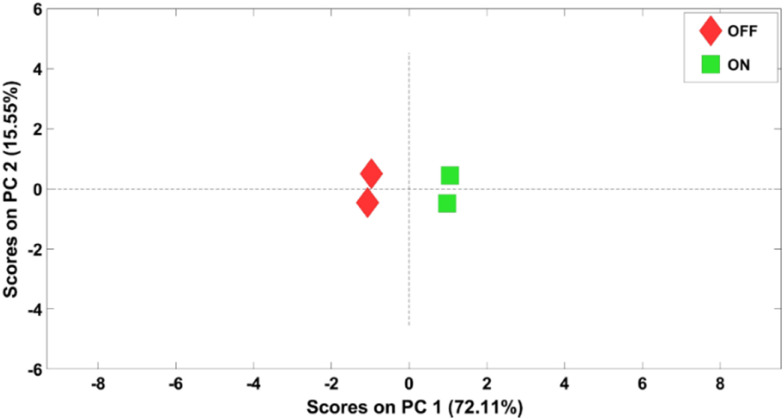
Score plot of the two first principal components on the PCA model of Ni 2p spectra: red spectra 1 and 3 (light OFF), green samples 2 and 4 (light ON).

The top spectrum in Fig. 2a shows the Ni 2p region recorded under 1 mbar H_2_O under light illumination of the catalyst. Interestingly, the Ni^0^ 2p peak disappears as light illuminates the catalyst and the main Ni 2p peak is broadened compared to that under dark conditions. As a result of the broadening in the Ni 2p peak, two new peaks can be fitted at 857.4 and 864.5 eV. Fig. 2b shows the difference spectra created by subtracting the spectrum recorded under dark conditions from the spectrum recorded under light illumination. These spectra show that when the light is turned ON, a peak disappears at 851.8 eV, while another broad peak appears at 857.4 eV with a satellite peak at 864.5 eV. However, no significant changes are observed in the BE positions of the peaks related to NiO and NiCO_3_. The peak at 851.8 eV can be assigned to Ni^0^, whereas the peaks centered at 857.4 and 864.5 eV can be attributed to NiOOH.^34^ NiOOH has seven satellite peaks, between 855 and 865 eV. However, their relative intensity is very small. Therefore, fitting all seven satellite peaks would not be a particularly attractive presentation due to the low peak intensities associated with NiOOH. Thus, the NiOOH peaks are fitted into two broad peaks centered at 857.4 and 864.5 eV. To justify our fitting and the reproducibility of the results, a PCA analysis was also carried out for the Ni 2p spectra for both cycles. The PCA results are shown as a score plot in Fig. 3 (spectrum numbers indicated in the inset of Fig. 2a; spectra # 1 and 2, Fig. S2;† spectra # 3 and 4). Based on the score plot, the first principal component (PC1) explains most of the variance in the data (72.11%). The spectra measured under dark conditions have negative and well-clustered scores (spectra 1 and 3, Fig. 2a and 3). The well-clustered negative scores for spectra no. 1 and 3 suggest that these spectra share common underlying components. Similarly, the spectra measured under light illumination have positive and well-clustered scores (spectra # 2 and 4, Fig. 2a and 3) suggesting common components between these two spectra. Additionally, since the PC1 scores of spectra 1 and 2 and spectra 3and 4 are different, this implies that there are distinct components or variations between spectra 1 and 2, as well as between spectra 3 and 4. The PCA supports the conclusion that there are distinct differences in the Ni 2p spectra recorded under light and dark conditions and is used to identify patterns and variations within the Ni 2p XPS spectra, confirming the reversibility of the Ni 2p spectra under light illumination and in the dark.

**Fig. 4:**
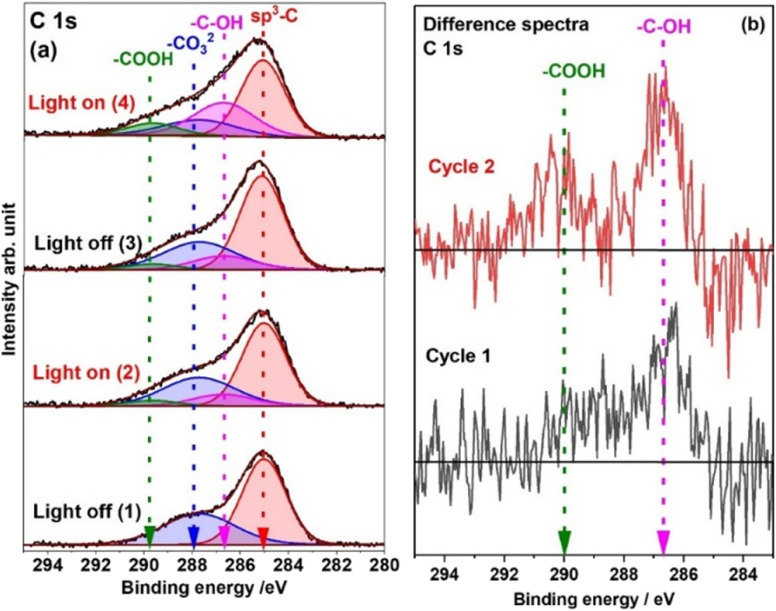
(a) C 1s spectra recorded under 1 mbar H_2_O with light illumination ON and OFF. Here two cycles are shown in the dark and under light illumination (spectra 1 and 2 belong to cycle 1, while spectra 3 and 4 belong to cycle 2). (b) Difference spectra of C 1s (black and red color spectra represent cycles 1 and 2, respectively); these spectra are obtained by subtracting the dark-condition recorded spectrum from the spectrum acquired under light illumination.

Fig. 4 illustrates the C 1s spectra recorded under 1 mbar H_2_O with light ON and OFF. As mentioned in Fig. 1b, the peak at 285 is identified as adventitious carbon, whereas the peak at 288 eV is related to the NiCO_3_ carbonate.^32^ The peak shape and position of the carbon spectra acquired under 1 mbar H_2_O pressure and dark conditions are very similar to the UHV spectra (Fig. 1b), *i.e.*, H_2_O does not have any substantial effect on the carbon peak without light illumination. However, a shift of 0.4 eV towards lower BE is observed for the carbonate peak compared to the UHV spectra. The water adsorption on the surface of the catalyst accounts for the shift.^40,41^ As soon as the light is turned ON, the C 1s region changes significantly. To identify the changes, the C 1s spectrum recorded under dark conditions is subtracted from the one recorded under light illumination. The subtracted spectra are shown in Fig. 4b. The C 1s difference spectrum shows two peaks at 286.7 eV and 289.7 eV. The 286.7 eV peak is assigned to the –C–OH bond^42–44^ and the 289.7 eV peak to the –COOH bond.^45,46^ The peak BE position for –C–OH is a little higher than the reported BE position for –C–O.^43,44^ The –C–OH group is formed as a result of hydrogen ion adsorption on carbonate.^18^ This means that the carbon in –C–OH is also attached to two oxygen atoms (=O and –O^−^). Due to the high electronegativity of oxygen attached to this carbon, C 1s shifts its BE towards the higher energy side. When the light was switched OFF after the first illumination cycle, the C 1s peaks did not change, indicating that the surface –C–OH and –COOH groups remained stable once they formed. However, during the second illumination cycle, when the solar light was turned ON again, the intensity of the C 1s peak associated with –C–OH (286.7 eV) and –COOH (289.7 eV) increased more than in the first illumination cycle. To further support the findings of the C 1s fitting, data analysis by PCA was also carried out. The outcomes of the PCA are shown in Fig. S3 of the ESI.† Spectra # 1 and 2 as well as 3 and 4 exhibit discernible variances in their score values, indicative of distinct compositional attributes (spectrum numbers indicated in the inset of Fig. 4 and S3†). This signifies that the C 1s spectral features significantly change when the solar simulator is switched ON. The same can be confirmed in the difference spectra shown in Fig. 4b. Conversely, spectra 2 and 3 demonstrate a pronounced resemblance in their compositional features. Analogously, spectra 1 and 2, as well as spectra 3 and 4, have different components but spectra 2 and 3 have the same components. These PCA findings serve to substantiate the validity of the analysis and fitting procedures for the C 1s XPS spectra.

**Fig. 5:**
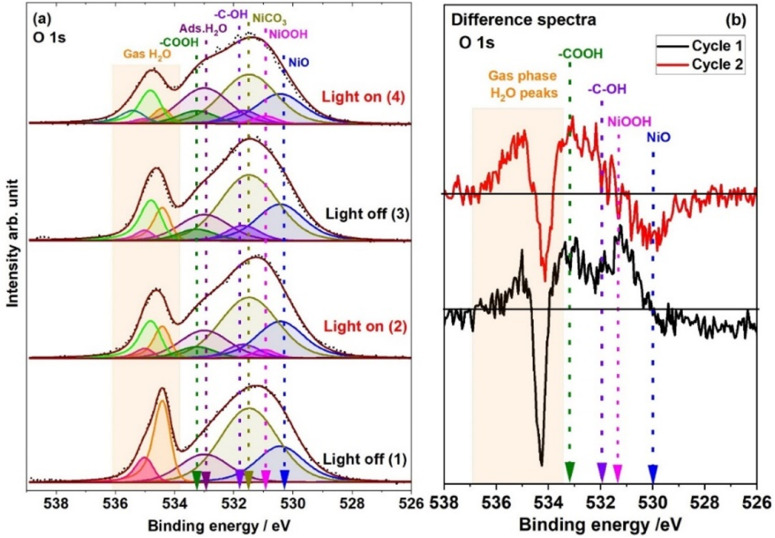
(a) O 1s spectra recorded under 1 mbar H_2_O with light illumination ON and OFF. Here two cycles are shown in the dark and under light illumination (spectra 1 and 2 belong to cycle 1 and spectra 3 and 4 belong to cycle 2). (b) O 1s difference spectra; (black and red color spectra represent cycles 1 and 2, respectively) derived by subtracting the spectrum acquired in the dark from the one acquired during light illumination. These O 1s spectra are fitted based on the peaks observed in the Ni 2p spectra (Fig. 2a), C 1s spectra (Fig. 4) and O 1s difference spectra (Fig. 5b) and PCA analysis of the O 1s spectra (Fig. S4†).

Fig. 5 shows the XPS O 1s spectra recorded under 1 mbar H_2_O vapour and with and without light illumination. The O 1s peak at 530.2 eV is associated with NiO, and the peak at 531.4 eV is associated with NiCO_3_.^47^ Meanwhile, the peak at 533 eV is associated with adsorbed water molecules.^48–50^ When the solar simulator light was switched ON, three new peaks were observed at 531.0, 531.7 and 533.2 eV, which are associated with NiOOH, the –C–OH group and –COOH on the catalyst surface, respectively.^42,51^ The presence of these three peaks is also evident in the difference spectra depicted in Fig. 5b which are assigned based on the peak intensity changes in the C 1s spectra in Fig. 4. When the light was switched off after the first light illumination cycle, the O 1s peaks associated with –C–OH and –COOH remained. However, the O 1s peak related to NiOOH disappeared. In the second illumination cycle, the O 1s intensity associated with –COOH and –C–OH groups increased compared to the first illumination cycle, and the peak associated with NiOOH reappeared. ESI† Fig. S4 elucidates the outcomes of PCA for the O 1s electron binding energies. Evidently, spectra 1 and 2 as well as 3 and 4 have different components based on their different score values, whereas spectra 2 and 3 demonstrate a pronounced resemblance in their compositional features (spectrum numbers indicated in the inset of Fig. 5 and S4†).

Water molecules present in a gas phase between the surface of the sample and the analyzer cone interact with NiO and NiCO_3_ on the catalyst's surface. This interaction leads to the observation of two distinct peaks in the gas phase water O 1s spectra at 534.4 and 535 eV (in Fig. 5 as orange and red color, respectively). It should also be noted that the gas phase water O 1s peaks at 534.4 and 535 eV evolve with the cycles and more than two components are needed to obtain a suitable fit once the light is switched ON and thereafter. The BEs of these gas phase peaks are very sensitive to the surface properties of catalysts.^52–54^ The BE changes in the gas phase spectra are directly correlated to the change in the work function of different surface species.^53^ Therefore, this shift in the BE of the gas phase spectra can be used to highlight the precise identification of the catalyst surface or interface changes that occur under the reaction/measurement conditions.^49,54^ In the first cycle in the dark, the two peaks at 534.4 and 535 eV are fitted for the gas phase water spectrum. This peak fitting is made under the assumption of interaction between gas phase water molecules and both NiO and NiCO_3_, each characterized by unique surface properties. As the light is turned ON, it is observed that the gas phase peaks become much broader. As a result of the peak broadening, another peak at 534.8 eV is needed to obtain a suitable fit (light green component), indicating that a different, transient catalyst surface with a different work function is exposed under light illumination compared to dark conditions. In the second illumination cycle, a similar trend in the gas phase oxygen spectra is observed with the gas phase spectra becoming even broader allowing a new peak centered at 535.4 eV to be fitted (dark cyan color). Therefore, it can be inferred that light illumination gives rise to transient changes in the surface of the catalyst, which has an electronically different nature from the surface when it is in the dark. This is in line with the observations from the Ni 2p and C 1s spectra, revealing the presence of NiOOH, –C–OH, and –COOH species. The broadening observed in the gas phase water O 1s peaks is attributed to the interaction between gas-phase H_2_O and NiOOH, –COOH, and –C–OH species present on the catalyst surface.^55,56^

### (c) Photocatalytic hydrogen evolution reaction (HER) measurements for Ni@Ni/NiCO_3_

**Fig. 6:**
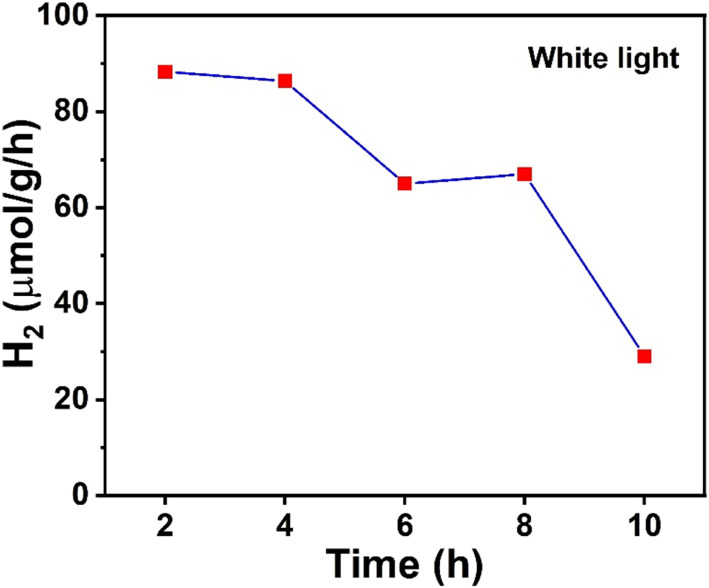
H_2_ yield measured by GC under white light on a Ni@NiO/NiCO_3_ catalyst. As the reaction time progressed, the amount of hydrogen produced decreased.

In assessing the photocatalyst's long-term stability, a ten hour HER measurement was conducted, and the hydrogen yield was measured every two hours using a GC. The GC analysis yielded valuable insights into the photocatalytic performance of the tested catalyst for hydrogen evolution. The results of water splitting experiments for Ni@Ni/NiCO_3_ are shown in Fig. 6. In the initial phase of the experiment, during a two hour exposure to white light, the catalyst demonstrated a remarkably high hydrogen production rate of 90 μmol g^−1^ h^−1^ without any sacrificial agents. This substantial rate indicates the efficient photocatalytic activity of the catalyst, making it a promising candidate for the HER. However, as the reaction progressed over a span of 10 hours, the observed H_2_ production rate exhibited a gradual and discernible decline. At its lowest, the rate reached 20 μmol g^−1^ per hour, presenting a significant reduction from the initial output.

## Discussion

**Fig. 7:**
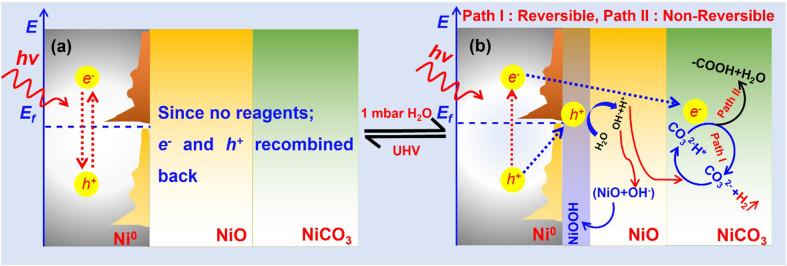
Photocatalytic HER mechanism under light illumination; under (a) UHV and (b) 1 mbar H_2_O pressure.

In the presence of light illumination and dark conditions, APXPS results provide detailed insight into the photocatalytic reaction occurring on the Ni@NiO/NiCO_3_ photocatalyst with the presence of 1 mbar H_2_O. The plausible reaction pathways implicated in the HER, as elucidated through analysis of the APXPS outcomes, are shown in Fig. 7 and the reaction steps are shown in eqn (1) to (4). The results of XPS measurement under UHV do not indicate any significant differences in the binding energies of Ni 2p, C 1s, and O 1s, or the peak ratios of NiO and NiCO_3_, irrespective whether the XPS spectra were recorded under light illumination or in the dark (Fig. 1). When the solar simulator is switched on, the collective oscillation of hot electrons takes place on Ni^0^ due to light absorption by the Ni nanoparticle SPR.^19,20^ In a vacuum, these so-called hot electrons could not be utilized, as there are no reactants present (Fig. 7). Consequently, the oscillating electrons eventually return to the ground state. Probably, the excited state is very short-lived, and the excited electron quickly returns to the ground state before being detected in XPS measurements under UHV conditions. On the other hand, NiO and NiCO_3_ are both large band gap materials (NiO with a 4 eV optical band gap and NiCO_3_ with a 3.6 eV band gap).^57–59^ Due to their significant band gaps, NiO and NiCO_3_ do not absorb visible light. Consequently, no significant changes were observed in the Ni 2p spectra of NiO and NiCO_3_ upon turning on the solar simulator, highlighting the observation that light illumination under UHV does not induce substantial changes in the electronic and chemical characteristics of the Ni@NiO/NiCO_3_ photocatalyst.

However, when the catalyst was exposed to light illumination under 1 mbar water vapor pressure, the electronic structure of Ni and carbonate underwent many interesting changes. When the solar simulator is switched ON, it is believed that Ni^0^ absorbs the light and generates holes and excites the electrons due to SPR (eqn (1)). The light absorption by Ni^0^ has been further substantiated by the UV-visible spectrum of the same catalyst, as measured by Talebi *et al.*, which shows a broad peak at 470 nm.^21^ This peak arises from the SPR effect of the Ni^0^ nanoparticles.^17,19,20^ Through APXPS data analysis, a hypothesis is formulated suggesting that the holes were transferred to NiO at the interface of Ni/NiO and trapped there.^60,61^ Furthermore, it is proposed that the oxidation process of H_2_O molecules progresses at NiO *via* an intermediate species, NiOOH, as delineated by eqn (3).^62^ A small decrease in the intensity of the O 1s peak related to NiO at 530 eV is observed in the difference spectra in Fig. 5b when the light is switched ON. The loss of O 1 s intensity related to the NiO peak (Fig. 5b) suggests the potential reversible transformation of NiO into NiOOH under light illumination conditions due to the oxidation of water molecules. NiO has been reported to be an effective photo-^63–65^ and electrocatalyst^62,66,67^ and co-catalyst for oxygen generation, attributed to its suitable oxygen generation potential and high efficiency in hole injection. Lin *et al.* observed using XAS during the oxygen evolution reaction (OER) that upon oxidation, the surfaces of NiO samples undergo a transformation to NiOOH.^68^ Furthermore, corroborating evidence from electrochemical studies^66,69,70^ and thermodynamic investigations^71^ supports that NiO is not stable under electro-^70,72^ and photochemical OERs.^71,73^ These studies support the transformation of NiO to Ni(OH)_2_ and NiOOH under both electro- and photochemical OERs.^69–72^ The transformation of NiO into different hydroxy species such as nickel hydroxide Ni(OH)_2_, NiOOH, or a mixture of Ni(OH)_2_/NiOOH during water oxidation reactions is a subject of debate.^70–73^ However, our *in situ* investigation confirms that during the photocatalytic OER, NiO undergoes reversible transformation into NiOOH, serving as an active site for the OER in photocatalysis.^73^ One of the most interesting aspects of the Ni 2p spectrum under 1 mbar H_2_O vapor is the vanishing of the Ni^0^ 2p peak under light illumination and reappearance in the dark (Fig. 2 and S2†). It is assumed that NiOOH accumulated then at the interface and covered the core of the catalyst. Therefore, the signal from the Ni^0^ 2p peak is lost under light illumination.

Another important component of this catalyst is NiCO_3_. Carbonates are regarded as promising candidates for improving photocatalytic oxidation and reduction;^58,74^ however, their potential role in these processes has not been fully explored.^59^ Usually, CO_2_, CO_3_^2−^ and HCO_3_^−^ are present in all aqueous media if pH > 4.8 However, the presence of CO_2_/HCO_3_^−^/CO_3_^2−^ is often neglected. Indeed, several studies have pointed out that the presence of HCO_3_^−^/CO_3_^2−^ in the system catalyzes photochemical and electrochemical water oxidation.^75–77^ Talebi *et al.* conducted density functional theory (DFT)-based first principles calculations and according to their results, NiCO_3_ is the favorable hydrogen adsorption site instead of NiO.^18^ In the same study, the Bader charge analysis indicated that the O in NiCO_3_ has a higher charge acceptor ability than the NiO. The present APXPS results and the previous DFT calculations signify that the hot electrons excited on Ni^0^ are transferred to the carbonate site where hydrogen ion reduction takes place. Indeed, the surface carbonates converted to bicarbonate like species [CO_3_^2−^(H)*, (eqn (4))] due to hydrogen ion adsorption. The transformation to CO_3_^2−^(H)* resulted in the manifestation of a peak in the C 1s spectra at 286.7 eV, attributed to the presence of CO_3_^2−^(H)* species. According to a study by Talebi *et al.* and observations using APXPS, these CO_3_^2−^(H)^*^ species may participate in two different reaction paths. These two different reaction paths are depicted in Fig. 7b and eqn (4a) and (4b). In path I, CO_3_^2−^(H)* reversibly converts back to –CO_3_^2−^ by generating H_2_ through the reduction of H^+^ ions. The resulting –CO_3_^2−^ then serves as a fresh active site for the subsequent reduction of new H^+^ ions (eqn (4a)). In irreversible path II, CO_3_^2−^(H)* is reduced to –COOH by the hydrogen ion produced under light illumination (eqn (4b)). The experiment was repeated twice, which creates differences in the C 1s spectra. When the solar light is turned ON again in the second illumination cycle, the intensity of the C 1s peak associated with –C–OH (286.7) increases more than that in the first illumination cycle (Fig. 4). This peak intensity can be explained by the fact that when the light is switched ON, more and more hydrogen ions are adsorbed onto carbonate. The intensity of the peak associated with –COOH at 289.7 eV also increases with each successive cycle. Indeed, during the HER, carbonates are converted into –COOH groups. Additionally, it is observed that the rate of carbonate conversion to –COOH groups is intimately correlated with the progression of the HER. The decline in hydrogen production observed in GC measurements can be attributed to the degradation of the carbonate into –COOH groups. Since carbonates are the preferred site for hydrogen ions according to DFT calculation,^18^ over time, these carbonates were also reduced into –COOH as the HER progressed (eqn (4b)), resulting in a decrease in the hydrogen production. However, the further participation of these –COOH groups in the photochemical reaction is out of the scope of the current study.

1 Ni^0^ + *hν* → Ni*

2 Ni^0^* → Ni^+^ + e^−^ + h^+^

3 NiO + H_2_O + h^+^ → NiOOH + H^+^

4 NiCO_3_  + H^+^ → NiCO_3_(H)*

4a
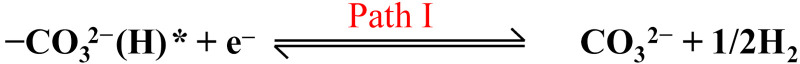
4b
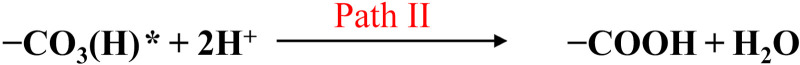


## Conclusions

Ambient pressure X-ray photoelectron spectroscopy provides an atomic-level understanding of the core–shell Ni@NiO/NiCO_3_ photocatalyst's active sites and transformation mechanisms under realistic conditions. The photocatalyst exhibits reversible structural and electronic changes when exposed to water vapor and solar simulator light. Light illumination under UHV conditions has no effect, but under *in situ* conditions, the hot electrons are transferred to NiCO_3_ for hydrogen ion reduction in the HER. Furthermore, NiO undergoes reversible oxidation to NiOOH. This work further highlights the role of carbonates in the reaction mechanism, rarely explored *via in situ* photocatalysis. However, additional dedicated studies will be required for a comprehensive understanding of the photocatalytic reduction mechanism of carbonates to –COOH as well as further participation of the –COOH group in photochemical reactions. The correlation between the carbonate degradation to –COOH and the diminishing hydrogen production rate over the period emphasizes the importance of understanding the photocatalyst's behavior over extended reaction times for future optimizations of the catalyst. In conclusion, APXPS can be utilized to understand the charge transfer dynamics for designing photocatalysts with improved charge separation and transfer capabilities, analyzing reaction intermediates to grasp the step-by-step progression of the photocatalytic reaction, and identifying potential bottlenecks or limiting factors in the reaction pathway for other photocatalytic processes such as CO_2_ reduction, organic pollutant purification, and N_2_ fixation as well.

## Conflicts of interest

There are no conflicts to declare.

## Supplementary Material

CY-014-D4CY00204K-s001
