# Supplementary material for: Solar light driven atomic and electronic transformations in a plasmonic Ni@NiO/NiCO3 photocatalyst revealed by ambient pressure X-ray photoelectron spectroscopy
Source: Catal Sci Technol. 2024 Apr 22;14(11):3029–40. doi: 10.1039/d4cy00204k (PMC11149490; doi:10.1039/d4cy00204k)
Supplement: CY-014-D4CY00204K-s001 [file CY-014-D4CY00204K-s001.pdf]

Electronic Supplementary Material (ESI) for Catalysis Science & Technology.
This journal is © The Royal Society of Chemistry 2024

Supporting Information

Solar Light Driven Atomic and Electronic Transformations in Plasmonic Ni@NiO/NiCO3 Photocatalyst revealed by Ambient Pressure X-ray Photoelectron Spectroscopy

Manoj Kumar Ghosalya,[1]* Parisa Talebi,[1] Harishchandra Singh,[1]* Alexander Klyushin,[2] Esko Kokkonen,[2] Mohammed Alaoui Mansouri,[1] Marko Huttula,[1] Wei Cao[1] and Samuli Urpelainen[1]*

1. Nano and Molecular Systems Research Unit, University of Oulu, FIN-90014, Finland.

2. MAX IV Laboratory, Lund University, Box 118, Lund, 22100, Sweden

*Corresponding authors: manoj.ghosalya@oulu.fi (MG), harishchandra.singh@oulu.fi (HS), and samuli.urpelainen@oulu.fi (SU)

Table of contents:

Figure S1: Ni 2p spectra recorded under UHV conditions with light illumination and dark conditions 2

Figure S2: The second cycle Ni 2p spectrum under dark and light illumination conditions 2

Figure S3: PCA analysis of C 1s spectra 3

Figure S4: PCA analysis of O 1s spectra 3

Table S1: Fitting Parameter for Ni 2p 4

Table S2: Fitting Parameter for C 1s 5

Table S3: Fitting Parameter for O 1s 6

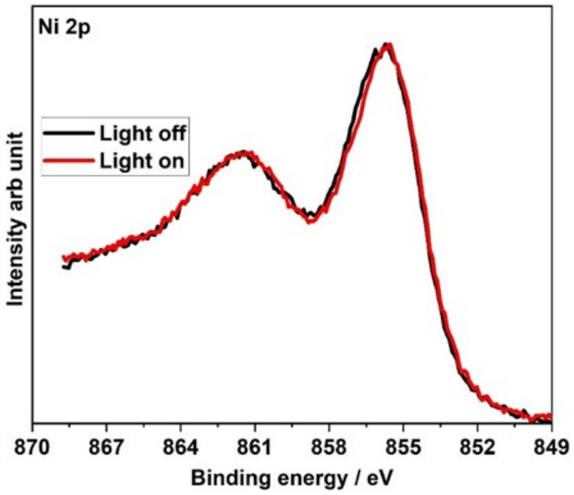

Figure S1: (a) Ni 2p spectra recorded under UHV conditions with light illumination and dark conditions. Spectra of Ni 2p are not significantly affected by light illumination.

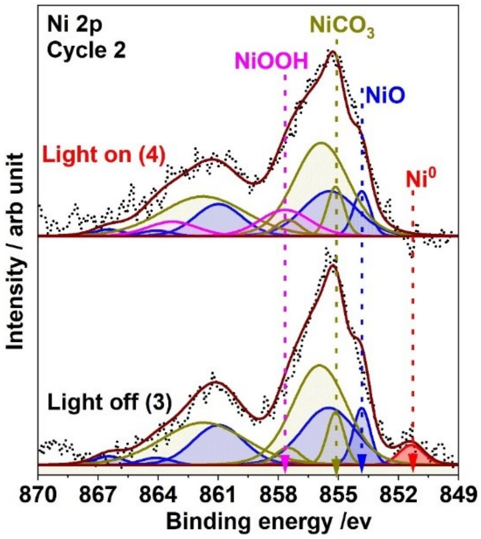

Figure S2:- The second cycle Ni 2p spectrum under dark and light illumination conditions with 1 mbar H2O is shown in Figure 3. In the main paper, we present the spectra for the first cycle. As can be seen from these spectra, the second cycle is like the first cycle, indicating that Ni 2p is reproducible.

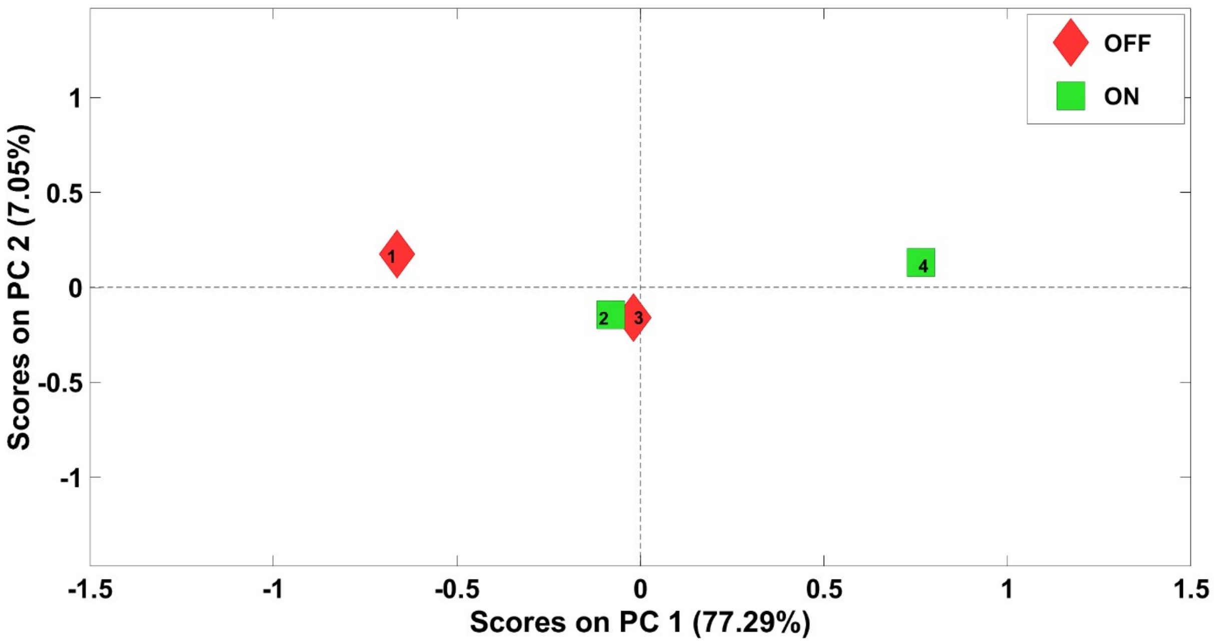

Figure S3: Score plot of the two first PCs on the PCA model of C 1s spectra: red spectra 1 and 3 (light OFF), green samples 2 and 4 (light ON)

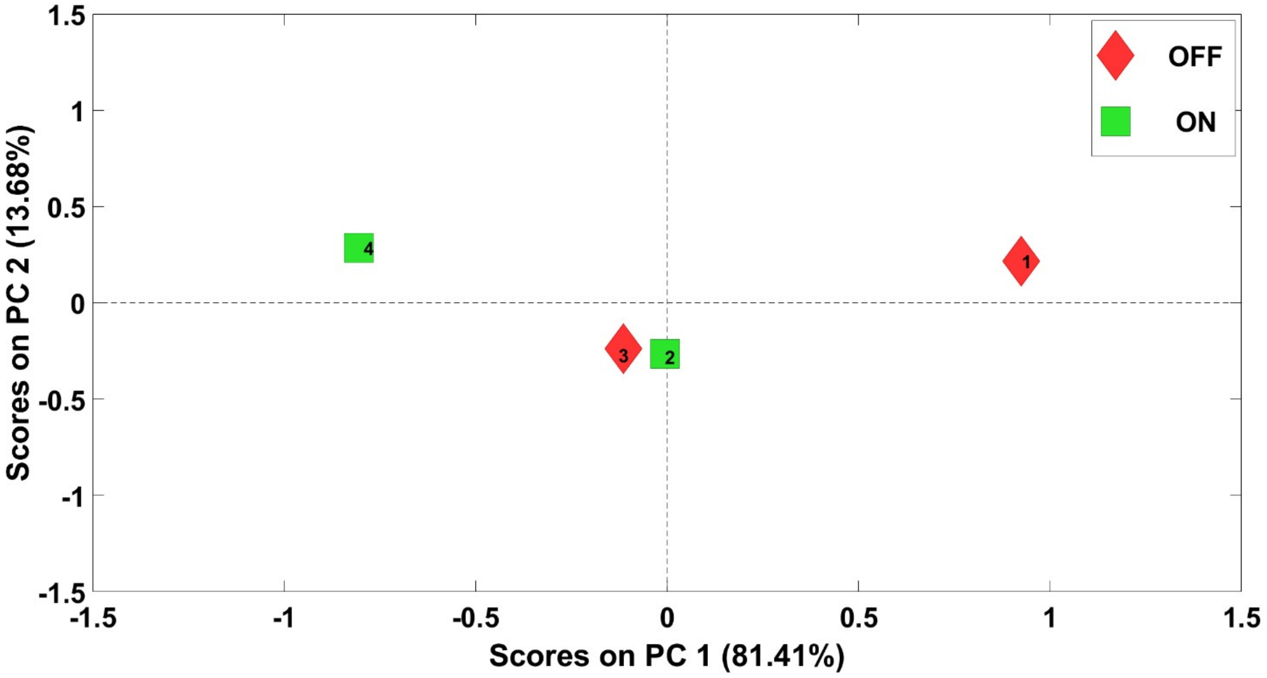

Figure S4: Score plot of the two first PCs on the PCA model of O 1s spectra: red spectra 1 and 3 (light OFF), green samples 2 and 4 (light ON)

Table S1._ Fitting Parameter for Ni 2p, of Ni@NiO/$NiCO_3$

	Ni^0			NiO					$NiCO_3$					Ni(OOH)	
**Peak no**	**1**	**2**	**3**	**1**	**2**	**3**	**4**	**5**	**1**	**2**	**3**	**4**	**5**	**1**	**2**
**Position (eV, UHV)**	852.1	855.77	860.12	854.12	855.62	861.06	864.62	866.75	855.20	856.2	858.20	860.50	862.0		
**Position (eV, 1 mbar H2O Light OFF)**	851.8	855.55	860.2	853.9	855.52	861.02	864.52	866.65	854.8	856.03	858.0	860.26	861.66		
**Position (eV, 1 mbar Light H2O ON)**				853.9	855.51	860.91	864.51	866.64	854.8	856.09	858.02	860.23	861.72	857.4	864.5
**FWHM (eV, UHV)**	1.5	2.3	2.2	1.2	3.2	4.2	1.9	2.55	1.26	2.36	1.35	2.15	5.2		
**FWHM (eV, 1 mbar H2O Light OFF)**	1.5	2.3	1.2	1.2	3.2	4.2	1.9	2.55	1.25	2.36	1.36	2.15	5.2		
**FWHM (eV, 1 mbar H2O Light ON)**				1.2	3.2	4.2	1.9	2.55	1.25	2.3	1.36	2.15	5.2	3.1	2.79

Table S2. Fitting Parameter for C 1s of Ni@NiO/$NiCO_3$

	**NiO**	**$NiCO_3$**	**-COOH**	**-C-OH**
**Position (eV, UHV**	285	288.4		
**Position (eV, 1 mbar H2O Light OFF)**	285	288	289.7	286.7
**Position (eV, 1 mbar H2O Light ON)**	285	288	289.7	286.7
**FWHM (eV, UHV)**	2.4	3.5		
**FWHM (eV, 1 mbar H2O Light OFF)**	2.2	3.5		
**FWHM (eV, 1 mbar H2O light ON)**	2.2	3.5	3.3	2.3

Table S3. Fitting Parameter for O 1s of Ni@NiO/$NiCO_3$

	NiO	$NiCO_3$	-COOH	NiOOH	-C-OH	Ads. H_2O		Gas phase peak		
							1	2	3	3
**Position (eV, UHV**	530.2	531.5								
**Position (eV, 1 mbar H2O Light OFF)**	530.2	531.4				533	534.4	535		
**Position (eV, 1 mbar H2O Light ON)**	530.2	531.4	533.2	531.0	531.7	533	534.4	535	534.8	535.4
**FWHM (eV, UHV)**	2.1	2.4								
**FWHM (eV, 1 mbar H2O Light ON)**	1.9	2.1				2	0.83			
**FWHM (eV, 1 mbar H2O light ON)**	1.9	2.2	1.95		2.1	2	0.75	0.85		0.1
